# Ketamine inhibits tumor necrosis factor secretion by RAW264.7 murine macrophages stimulated with antibiotic-exposed strains of community-associated, methicillin-resistant *Staphylococcus aureus*

**DOI:** 10.1186/1471-2172-12-11

**Published:** 2011-01-25

**Authors:** Thomas Spentzas, Rebekah KH Shapley, Carlos Acuna Aguirre, Elizabeth Meals, Lauren Lazar, Mark S Rayburn, Brett S Walker, B Keith English

**Affiliations:** 1 Department of Pediatrics, University of Tennessee Health Science Center, Memphis, TN, USA; 2 Children's Foundation Research Center at Le Bonheur Children's Hospital, Memphis, TN, USA; 3 Division of Pediatric Critical Care Medicine, Le Bonheur Children's Hospital, Memphis, TN, USA; 4 Department of Clinical Pharmacy, Le Bonheur Children's Hospital, Memphis, TN, USA; 5 Division of Pediatric Infectious Diseases, Le Bonheur Children's Hospital, Memphis, TN, USA

## Abstract

**Background:**

Infections caused by community-associated strains of methicillin-resistant *Staphylococcus aureus *(CA-MRSA) are associated with a marked and prolonged host inflammatory response. In a sepsis simulation model, we tested whether the anesthetic ketamine inhibits the macrophage TNF response to antibiotic-exposed CA-MRSA bacteria via its antagonism of N-methyl-D-aspartate (NMDA) receptors. RAW264.7 cells were stimulated for 18 hrs with 10^5 ^to 10^7 ^CFU/mL inocula of either of two prototypical CA-MRSA isolates, USA300 strain LAC and USA400 strain MW2, in the presence of either vancomycin or daptomycin. One hour before bacterial stimulation, ketamine was added with or without MK-801 (dizocilpine, a chemically unrelated non-competitive NMDA receptor antagonist), APV (D-2-amino-5-phosphono-valerate, a competitive NMDA receptor antagonist), NMDA, or combinations of these agents. Supernatants were collected and assayed for TNF concentration by ELISA.

**Results:**

RAW264.7 cells exposed to either LAC or MW2 in the presence of daptomycin secreted less TNF than in the presence of vancomycin. The addition of ketamine inhibited macrophage TNF secretion after stimulation with either of the CA-MRSA isolates (LAC, MW2) in the presence of either antibiotic. The NMDA inhibitors, MK-801 and APV, also suppressed macrophage TNF secretion after stimulation with either of the antibiotic-exposed CA-MRSA isolates, and the effect was not additive or synergistic with ketamine. The addition of NMDA substrate augmented TNF secretion in response to the CA-MRSA bacteria, and the addition of APV suppressed the effect of NMDA in a dose-dependent fashion.

**Conclusions:**

Ketamine inhibits TNF secretion by MRSA-stimulated RAW264.7 macrophages and the mechanism likely involves NMDA receptor antagonism. These findings may have therapeutic significance in MRSA sepsis.

## Background

Infections caused by community-associated strains of methicillin-resistant *Staphylococcus aureus *(CA-MRSA) present a major public health problem because of recent increases in the incidence of these infections [1,2]. In a 2007 report, the Centers for Disease Control concluded that *Staphylococcus aureus *is now the most important cause of serious and fatal infection in the United States [3]. The prototypical USA400 strain, MW2, (CDC nomenclature for this strain of MRSA) was first isolated in 1999 from a Midwest child with fatal CA-MRSA pneumonia [4]. In 2003, the prototypical USA300 CA-MRSA strain, LAC, was isolated from Los Angeles County patients with skin and soft tissue infections, severe pneumonia and sepsis. Recently, concerns about CA-MRSA infections were heightened after reports of severe invasive staphylococcal infections in some patients infected with the novel 2009 H1N1 influenza A virus [5,6].

CA-MRSA isolates express many virulence factors [7,8], including several cytolysins: α-toxin, γ-toxin, Panton-Valentine leukocidin (PVL), phenol-soluble modulins (PSMs), δ-toxin and, unlike traditional hospital-associated (HA-MRSA) isolates, may express superantigens such as TSST-1 [9]. These bacterial components can stimulate massive cytokine release and lead to septic shock, acute respiratory distress syndrome (ARDS) and death. It is likely that strategies designed to modulate the excessive and prolonged host inflammatory response could improve the outcome of fulminant MRSA infections.

Monocytes and macrophages play important roles in host defense against staphylococci and other pyogenic bacteria [10], but excessive systemic or local production of inflammatory mediators by macrophages could be deleterious in patients with severe staphylococcal infections. We previously reported that RAW264.7 murine macrophages exposed to any of a series of six pediatric clinical isolates of *S. aureus *(two CA-MRSA, two HA-MRSA, and two methicillin-susceptible strains) in the presence of daptomycin (vs. vancomycin) secreted less TNF and accumulated less inducible nitric oxide synthase (iNOS) protein [11]. Vancomycin is a cell-wall active antibiotic that triggers bacterial lysis; it is the antibiotic most commonly used to treat severe MRSA infections in children [12]. Daptomycin is a novel antibiotic that is rapidly bactericidal against staphylococci but does not appear to cause rapid bacterial lysis; the mechanism of its action is not certain but it is reported to trigger depolarization of the bacterial membranes and inhibition of both DNA and RNA synthesis [13,14]. The rapid lysis of staphylococci, streptococci and other pyogenic bacteria exposed to cell-wall active antibiotics such as beta-lactams and vancomycin results in exaggerated release of bacterial products and an augmented and potentially harmful host inflammatory response [15,16]. Therefore, optimal treatment of sepsis and other severe bacterial infections might include the use of antibiotics and/or other medications that blunt the host inflammatory response and dampen the cytokine cascade [16].

Ketamine is one of the recommended anesthetics in pediatric septic shock [17-19], which is frequently caused by staphylococci [12,20]. The reasoning for ketamine's use in staphylococcal septic shock is its blood pressure supporting effect. It increases cardiac output and blood pressure, possibly via a catecholamine release mechanism [17,21]. Some data suggest that ketamine has anti-inflammatory effects [22-25]. For example, it has been reported that ketamine suppresses macrophage TNF secretion in response to Gram-negative bacterial LPS *in vivo *and *in vitro *[22,23,25]. There is also one report that ketamine suppresses TNF production by human whole blood *in vitro *after exposure to staphylococcal enterotoxin B [24]. The mechanisms responsible for the anti-inflammatory effects of ketamine are not known [22-25].The present study examined the hypothesis that ketamine could suppress macrophage TNF production in response to whole bacteria, in this case clinical isolates of methicillin-resistant *Staphylococcus aureus *(MRSA). Given the important role of TNF in sepsis [26-29], and the importance of staphylococcal sepsis in children, such suppression could have a therapeutic impact.

Although membrane-bound Toll-like receptors (TLR2 and TLR4) are essential for lipopolysaccharide (LPS)-induced TNF production [30], this is not the case for *Staphylococcus aureus*. Because *S. aureus *is able to "attack" or form pores in macrophages, TNF secretion occurs even in the absence of TLR 2 and TLR4 sensors (possibly via Nod1 and Nod2, intracytoplasmic sensors of peptidoglycan-derived muropeptides) [31]. Therefore, another mechanism independent of Toll-like receptors must exist for ketamine's anti-inflammatory action, at least in staphylococcal infections.

We also tested the effects of two chemically unrelated NMDA receptor antagonists, the anti-convulsant MK-801 (dizocilpine) [32,33], a non-competitive inhibitor of NMDA receptors, and APV (D-2-amino-5-phosphono-valerate), a competitive NMDA receptor antagonist [34,35], as well as the NMDA substrate itself, on macrophage TNF secretion in response to antibiotic-treated CA-MRSA bacteria.

## Methods

### Bacteria

For these studies, we utilized two well-characterized clinical isolates: LAC (Los Angeles County), representative of the USA300 group of organisms and closely related to the dominant CA-MRSA clone associated with soft tissue infections and serious invasive disease in the Memphis area [1], and MW2, a clinical isolate from a midwestern child with fatal CA-MRSA sepsis [4], representative of the USA400 group of organisms that constitute the other main lineage of CA-MRSA isolates in the United States.

Bacteria were grown to late logarithmic phase at 37°C in tryptic soy broth (Becton Dickinson and Co., Sparks, MD) and washed three times in endotoxin-free phosphate-buffered saline. Concentrations were determined by colony counts. A range of concentrations of bacteria (10^5^-10^7 ^CFU/mL) was studied, based upon our previously published data with other CA-MRSA strains [11] and our preliminary experiments using LAC and MW2 (data not shown).

Minimum inhibitory concentrations (MICs) for these strains were determined by the microbiology laboratory at Le Bonheur Children's Hospital using the E-test method: both strains were fully susceptible to vancomycin and daptomycin (LAC: MIC vancomycin 1.0 μg/mL; daptomycin 0.75 μg/mL; MW2: MIC vancomycin < 0.5 μg/mL; daptomycin 0.75 μg/mL).

### Cell culture

RAW264.7 murine macrophage-like cells were purchased from the ATCC and cultured in Dulbecco's modified Eagle's medium (Mediatech Inc., Herndon, VA) supplemented with 10% fetal bovine serum (HyClone, Logan, UT) and 2 mM glutamine (GIBCO, Carlsbad, CA). Experiments were done in 24-well tissue culture plates (Becton Dickinson, Lincoln Park, NJ) with 1 × 10^6 ^cells per well.

Either vancomycin or daptomycin was added to the cell cultures immediately before the addition of live staphylococci (10^5^-10^7 ^CFU/mL). Cells were then incubated for 18 hours. Daptomycin was obtained from Cubist Pharmaceuticals (Lexington, MA). Vancomycin was purchased via the Department of Pharmacy at Le Bonheur Children's Hospital (LBCH) from Hospira (Lake Forest, IL). Clinically achievable concentrations of each of the antibiotics, as previously tested in our laboratory [11], were used (20 μg/mL).

These experiments were repeated in parallel in the presence of ketamine (100 μM) and/or MK-801 (dizocilpine, 150 μM), APV (D-2-amino-5-phosphonovalerate, 300 μM ("low") or 3 mM ("high"), or NMDA (30 μM). The modulation of MRSA-stimulated macrophage TNF production by ketamine was subsequently examined also at a range of concentrations of 10 μΜ, 50 μΜ, 100 μΜ and 150 μΜ. The selected concentration (100 μM) is based on the achievable anesthetic concentrations [36-39] and on the pre-existing literature related to ketamine's TNF suppressive effect on murine macrophage models when stimulated by LPS [23-25,27]. The concentrations for the other factors were selected from the available literature, MK-801 [40-42], APV and NMDA [32] have previously been studied in cell culture models and have been shown to not cause cytotoxicity at the tested concentrations. Ketamine and/or MK-801 or APV or NMDA were added to the macrophage cultures one hour prior to bacterial challenge. The source of ketamine was Ketalar^®^, a racemic mixture (1:1) of optically active isomers (R and L) of this drug, purchased from the LBCH pharmacy. Emphasis in the experiment was placed on correlation with the clinical situation; thus racemic ketamine, the most commonly clinically used product, was selected. Dizocilpine (MK-801), APV and NMDA were purchased from Sigma Chemical Co. (St. Louis, MO).

After incubation, cell-free supernatants were collected and assayed for TNF concentrations by using a solid-phase sandwich enzyme-linked immunosorbent assay as specified by the manufacturer (eBioscience, San Diego, CA). TNF is a key cytokine produced by macrophages during MRSA stimulation. In our preliminary studies, we also measured secretion of other cytokines and found that IL-1, IL-6, and NO secretion were strongly correlated with TNF secretion in response to these bacteria (r^2 ^= 0.84, 0.87 and 0.93, respectively). We focused on TNF secretion for these studies.

The tested concentrations of vancomycin, daptomycin, ketamine, MK-801, APV, and NMDA had no effect on the viability of the RAW264.7 cells, as determined by visual inspection of the monolayer, low power microscopic inspection of the monolayer and exclusion of 0.2% trypan blue dye.

For the single comparison experiments (ketamine or MK 801 or APV), TNF secretion measurements were validated with an average of at least three well replicates and each of the experiments was repeated at least three times (a total of at least nine samples). The four preliminary runs and all the exposures (total of 16) where the inocula were different from 10^5 ^to10^7 ^CFUs/mL at the verifying colony count were excluded from the final analysis. Experiments with different exposure times (6, 10, 14, 24 hours) were conducted to determine whether the inhibition increased over time. In the multiple comparison experiments (ketamine and MK 801 synergistic action), TNF was measured from at least four well replicates. All experiments were performed separately for LAC and for MW2 MRSA strains. There is an intrinsic experimental variation of absolute values of TNF production (up to 25%) because of cell culture and macrophage growth characteristics.

### Data analysis

The design was composed of factorial multiple measurements and the results were analyzed according to a mixed linear model, (GLIMMIX) SAS 9.2 (SAS Institute, Cary, NC) and R 2.9.1 and ggplot2 software. We set pre-planned (*a priori) *contrasts, i.e., we set all our comparisons in advance of multiple setting experiments. Significant differences were presumed at a probability value of *p *< 0.05. The results were graphed using error bars with 95% confidence intervals. Differences in the means were estimated either with asymptotic techniques for normally distributed data or bootstrapping techniques for non-normally distributed data.

## Results

### CA-MRSA strains MW2 and LAC stimulated less TNF secretion by RAW264.7 murine macrophages in the presence of daptomycin than in the presence of vancomycin

**Figure 1:**
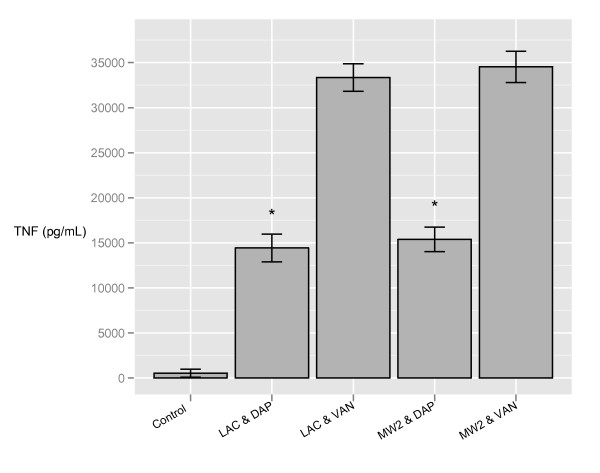
**The CA-MRSA isolates LAC (USA300) and MW2 (USA400) stimulated less TNF secretion by RAW264.7 murine macrophages when exposed to daptomycin (DAP) than when exposed to vancomycin (VAN)**. LAC or MW2 were added to RAW264.7 cells at a final concentration of 10^5 ^to 10^7 ^CFU/mL (retrospective confirmation) in the presence of either vancomycin or daptomycin at 20 μg/mL. Cells were incubated for 18 hours; supernatants were collected and analyzed for TNF content by an enzyme-linked immunosorbent assay (ELISA). Results are depicted as means with 95% confidence intervals shown as "error bars" (See Methods). The "*" indicates significance at *p *< 0.05. Control represents the mean TNF macrophage production by macrophages not stimulated with bacteria.

As previously observed with two USA300 CA-MRSA strains isolated from Memphis children with invasive staphylococcal infections [11], macrophages exposed to either of the two prototypical CA-MRSA strains studied (the USA300 strain, LAC, or the USA400 strain, MW2) secreted significantly less TNF in the presence of daptomycin as compared with vancomycin (more than 50% reduction in each strain; Figure 1). Macrophage TNF secretion in response to MW2 was 34,535 ± 1,536 pg/mL in the presence of vancomycin and 15,377 ± 1,267 pg/mL in the presence of daptomycin, a reduction of 55%, significant at *p *< 0.05. Similarly, macrophage TNF secretion in response to LAC in the presence of vancomycin was 33,345 ± 1,535 pg/mL, and 14,432 ± 1,536 pg/mL in the presence of daptomycin, a reduction of 57%, significant at *p *< 0.05. We previously reported similar findings in six *S. aureus *clinical isolates (including two pediatric CA-MRSA isolates of the USA300 group), suggesting that this effect of daptomycin is conserved in many different *S. aureus *isolates.

### Ketamine inhibited TNF secretion by murine macrophages stimulated with CA-MRSA isolates in the presence of antibiotics

**Figure 2:**
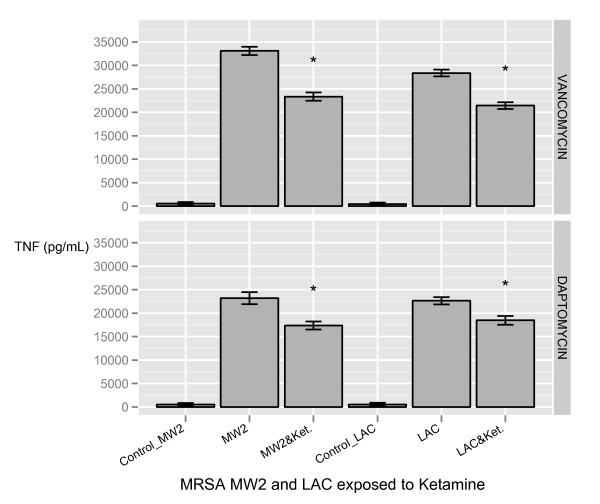
**Ketamine inhibited TNF secretion by RAW264.7 murine macrophages stimulated with antibiotic-treated CA-MRSA isolates LAC and MW2**. LAC or MW2 were added to RAW264.7 cells at a final concentration of 10^5 ^to 10^7 ^CFU/mL (retrospective confirmation) in the presence of either vancomycin at 20 μg/mL (upper panel) or daptomycin at 20 μg/mL (lower panel). One hour prior to stimulation, ketamine (100 μM) was added to the indicated wells. Cells were then incubated for 18 hours; supernatants were collected and analyzed for TNF content by ELISA. Results are depicted as means with 95% confidence intervals shown as "error bars" (See Methods). The "*" indicates significance at *p *< 0.05. Control represents the mean TNF macrophage production by macrophages not stimulated with bacteria.

The addition of ketamine (100 μΜ) to macrophage cell cultures inhibited TNF secretion in response to vancomycin- or daptomycin-exposed CA-MRSA isolates (Figure 2). The effect was similar on both strains, LAC and MW2, in the presence of vancomycin (upper panel) or daptomycin (lower panel).

In the initial experiments we analyzed the effect of one hour pre-incubation with ketamine on the macrophage response to vancomycin-exposed CA-MRSA bacteria (MW2 and LAC). In response to vancomycin-exposed MW2, pre-incubation with ketamine reduced macrophage TNF secretion by approximately 29% (*p *< 0.05), i.e., from 33,085 ± 867 pg/mL to 23,347 ± 862 pg/mL. Pre-incubation with ketamine led to a similar reduction (25%; *p *< 0.05) in macrophage TNF secretion response after stimulation with vancomycin-exposed LAC (from 28,365 ± 735 pg/mL to 21,432 ± 736 pg/mL).

We next studied the effect of ketamine pre-incubation on macrophage TNF secretion after stimulation with daptomycin-exposed MW2 or LAC. Once again, the addition of ketamine resulted in significant inhibition of macrophage TNF secretion in response to MW2 (23,185 ± 1,267 pg/mL to 17,354 ± 853 pg/mL, a reduction of approximately 25%; *p *< 0.05) or LAC (approximately 18% reduction, *p *< 0.05; Figure 2). Adding ketamine after the MRSA inocula did not alter the response.

### The NMDA inhibitor MK-801 (dizocilpine) inhibited macrophage TNF secretion after stimulation with antibiotic-exposed CA-MRSA strains

**Figure 3:**
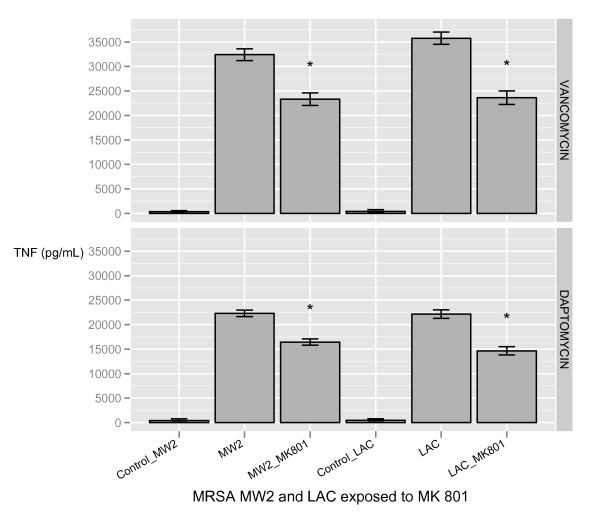
**MK-801 inhibited TNF secretion by RAW264.7 murine macrophages stimulated with antibiotic-treated CA-MRSA isolates LAC and MW2**. LAC or MW2 were added to RAW264.7 cells at a final concentration of 10^5 ^to 10^7 ^CFU/mL (retrospective confirmation) in the presence of either vancomycin at 20 μg/mL (upper panel) or daptomycin at 20 μg/mL (lower panel). One hour prior to stimulation, MK-801 (150 μΜ) was added to the indicated wells. Cells were then incubated for 18 hours; supernatants were collected and analyzed for TNF content by ELISA. Results are depicted as means with 95% confidence intervals shown as "error bars" (See Methods). The "*" indicates significance at *p *< 0.05. Control represents the mean TNF production by macrophages not stimulated with bacteria.

Pre-incubation of RAW264.7 cells for one hour with the NMDA receptor antagonist, MK-801 (150 μΜ), also inhibited TNF secretion by these cells after stimulation with antibiotic-exposed CA-MRSA strains (MW2 or LAC, Figure 3). In response to stimulation with MW2 in the presence of vancomycin, pre-incubation with MK-801 significantly inhibited TNF secretion by these cells, i.e., from 32,407 ± 1,188 pg/mL to 23,337 ± 1,272 pg/mL (approximately 28% reduction; *p *< 0.05, Figure 3, upper panel). MK-801 also inhibited macrophage TNF secretion in response to vancomycin-exposed LAC, causing a 34% reduction (Figure 3, upper panel).

Pre-incubation with MK-801 also significantly inhibited macrophage TNF secretion in response to daptomycin-treated MW2 or LAC (Figure 3, lower panel). In response to stimulation with MW2 in the presence of daptomycin, pre-incubation with MK-801 inhibited TNF secretion by these cells by approximately 26% (from 22,305 ± 648 pg/mL to 16,437 ± 642 pg/mL, *p *< 0.05). MK-801 inhibited macrophage TNF secretion in response to daptomycin-exposed LAC by approximately 33% (from 22,164 ± 864 pg/mL to 14,647 ± 832 pg/mL, *p *< 0.05).

### No additive or synergistic inhibition of macrophage TNF secretion is observed after pre-incubation with ketamine plus MK-801

**Figure 4:**
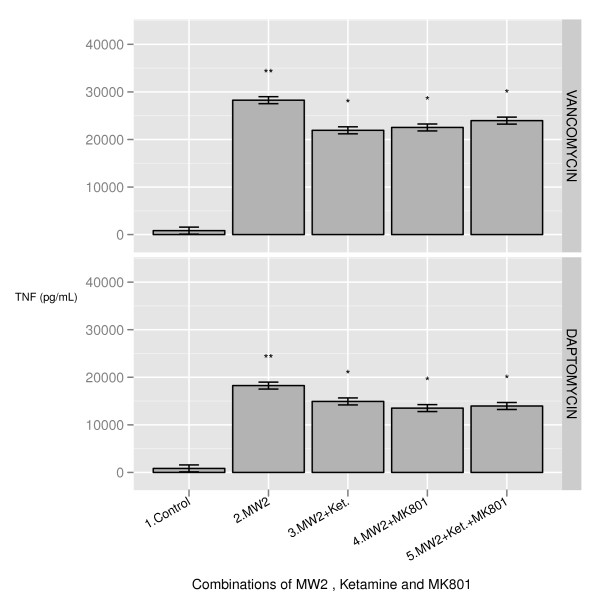
**No additive or synergistic effects of combinations of MK-801 and ketamine on macrophage TNF secretion, in response to antibiotic-treated CA-MRSA strain MW2, were seen**. Bacteria were added at a final concentration of 10^5 ^to10^7 ^CFU/mL (retrospective confirmation) in the presence of either vancomycin at 20 μg/mL (upper panel) or daptomycin at 20 μg/mL (lower panel). One hour prior to stimulation, either ketamine at 100 μM, MK-801 at 150 μΜ, or both were added to the indicated wells. Cells were then incubated for 18 hours; supernatants were collected and analyzed for TNF content by ELISA. Lane 1 (control) represents the mean TNF production by macrophages not stimulated with bacteria. The mean includes wells exposed to ketamine, MK-801, both ketamine and MK-801, and neither. In the absence of bacteria, TNF secretion was minimal and was not affected by ketamine and/or MK-801. Lanes 2 -5 depict mean TNF secretion by macrophages exposed to vancomycin-treated MW2 alone (lane 2), MW2 + ketamine (lane 3), MW2 + MK-801 (lane 4), or MW2 + ketamine + MK-801 (lane 5). The "*" on bars 3, 4, 5 indicates that they are statistically different (*p *< 0.05) from bars 1 and 2. The "**" on bar 2 indicates significantly higher TNF production (*p *< 0.05).

Pre-incubation of RAW264.7 cells with combinations of MK-801 and ketamine did not affect the magnitude of inhibition of macrophage TNF secretion observed in the presence of ketamine (or MK-801) alone. Figure 4 depicts results for macrophages stimulated with vancomycin- or daptomycin-exposed MW2; responses to antibiotic-exposed LAC were similar (data not shown).

### NMDA augments macrophage TNF secretion in response to antibiotic-treated CA-MRSA bacteria: both ketamine and a competitive NMDA receptor antagonist, APV, block this effect

**Figure 5:**
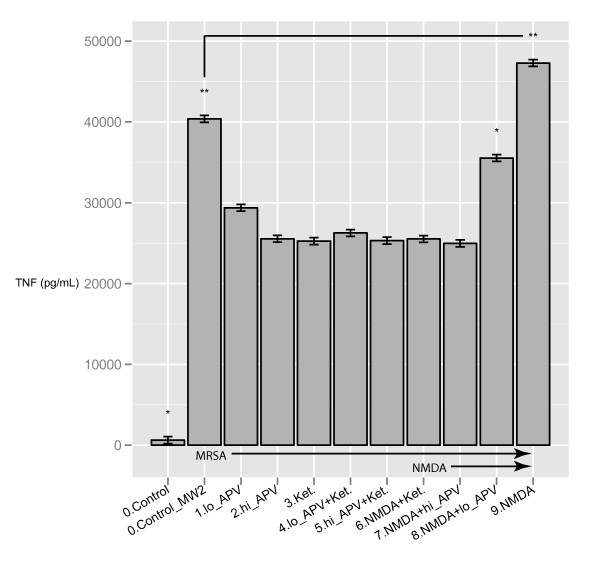
**APV inhibited and NMDA augmented TNF secretion by RAW264.7 murine macrophages stimulated with the antibiotic-treated CA-MRSA isolate, MW2**. Bacteria were added at a final concentration of 10^5 ^to10^7 ^CFU/mL (retrospective confirmation) in the presence of vancomycin at 20 μg/mL. One hour prior to stimulation, APV ("low" concentration of 300 μM or "high" concentration of 3 mM), ketamine (100 μM), or NMDA (30 μM) were added, alone or in combination, as indicated. Cells were then incubated for 18 hours; supernatants were collected and analyzed for TNF content by ELISA. The control lane represents the mean TNF macrophage production by macrophages not stimulated with bacteria. The mean includes wells exposed to APV, ketamine, or NMDA alone or in combination. In the absence of bacteria, TNF secretion was minimal and was not affected by APV, ketamine, or NMDA. Lanes 0-9 depict mean TNF secretion by macrophages exposed to vancomycin-treated MW2 alone (lane 0) or in the presence of the indicated concentrations of APV, ketamine, and/or NMDA (lanes 1-9). TNF secretion was reduced by approximately 30-40% when macrophages were pre-incubated with APV, ketamine, or APV + ketamine (lanes 1-5). The magnitude of inhibition by ketamine and high-dose APV was similar and there were no additive or synergistic effect observed with combinations of ketamine and APV. Addition of NMDA (30 μΜ) led to a substantial increase in the amount of TNF secreted in response to the MW2 strain (lane 9), and this augmented response was blocked by both APV and ketamine. The "*" on "0.Control" and "8.NMDA+lo_APV" bars indicates significance at *p *< 0.05. The "**" on "0.Control_MW2" and "9.NMDA" bars indicates differences between the pretreated wells, and that TNF production after MRSA stimulation with NMDA substrate (9.NMDA) is significantly higher than that at the baseline MRSA stimulation (0.Control_MW2) at *p *< 0.05.

We further examined the role of NMDA receptors in modulating the macrophage TNF response to the CA-MRSA bacteria by studying the effects of a competitive NMDA receptor antagonist, APV, and the effects of the NMDA substrate itself (Figure 5). We found that APV (at either 300 μM or 3 mM) also inhibited macrophage TNF secretion in response to vancomycin-exposed MW2 (*p *< 0.05, Figure 5). The magnitude of the inhibition was comparable to that observed with either ketamine or MK-801 (and, as in the case of MK-801, was not additive or synergistic with ketamine). Furthermore, the addition of the NMDA substrate (30 μM) resulted in a marked augmentation of the macrophage TNF response to the antibiotic-treated CA-MRSA bacteria (*p *< 0.05), and this effect was blocked by ketamine and by the competitive NMDA receptor antagonist, APV (Figure 5).

### Inhibition of macrophage TNF secretion is observed across a range of ketamine concentrations and throughout the incubation period

**Figure 6:**
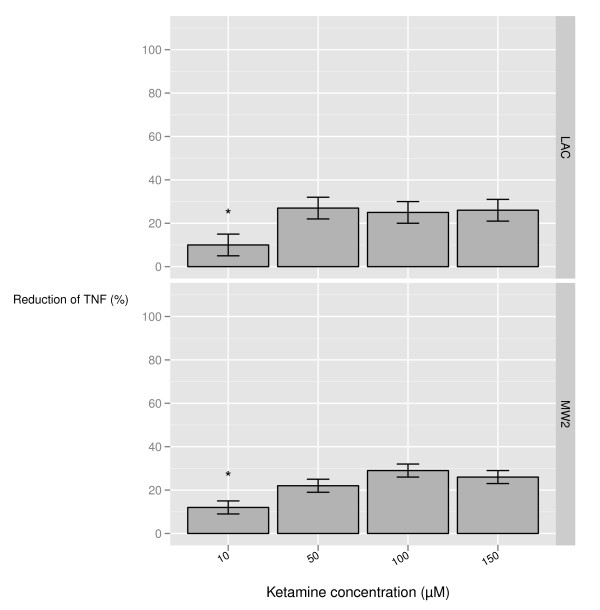
**TNF suppression by ketamine was tested at concentrations of 10 μM, 50 μM, 100 μM and 150 μM in the presence of vancomycin at 20 μg/mL and RAW264 macrophage stimulation with either LAC (upper panel) or MW2 (lower panel) CA-MRSA strains**. Bacteria were added at a final concentration of 10^5 ^to10^7 ^CFU/mL (retrospective confirmation) in the presence of vancomycin at 20 μg/mL. The inoculation time was 18 hours. Results are depicted as percentile reduction with 95% confidence intervals shown, i.e., the percent of TNF reduction that occurs after the specific concentration of ketamine was added.

We next studied the effects of a range of concentrations of ketamine and found that inhibition of macrophage TNF secretion in response to vancomycin-exposed LAC or MW2 was consistently observed at concentrations of ketamine at the lowest concentration tested (10 μM) and was greater at concentrations of 50 -150 μM (Figure 6).

**Figure 7:**
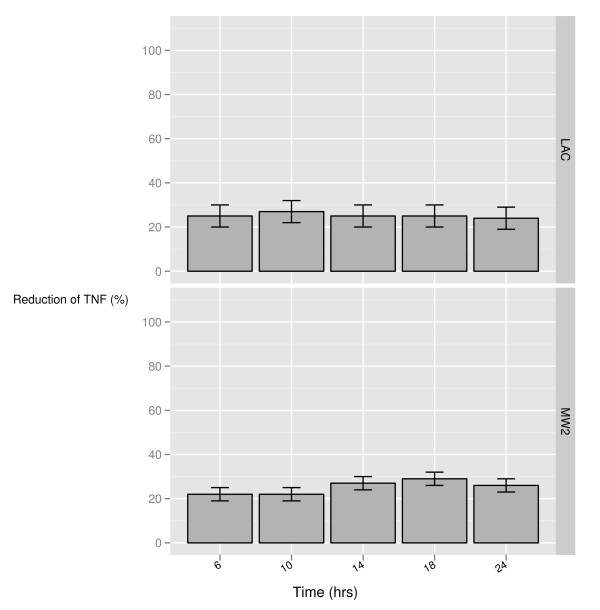
**TNF suppression by ketamine was tested after 6, 10, 14, 18 and 24 hours exposure in the presence of vancomycin at 20 μg/mL and RAW264 macrophage stimulation with either LAC (upper panel) or MW2 (lower panel) CA-MRSA strains**. Bacteria were added at a final concentration of 10^5 ^to10^7 ^CFU/mL (retrospective confirmation) in the presence of vancomycin at 20 μg/mL. The ketamine concentration was 100 μM. Results are depicted as percentile reduction with 95% confidence intervals, i.e., the percent of TNF reduction at the specific exposure time that occurs in comparison to inoculation without ketamine at the same time. The "*" indicates statistically significant difference at *p *< 0.05.

We also examined the kinetics of inhibition of macrophage TNF secretion by incubating RAW264.7 cells for 6, 10, 14, 18 and 24 hours after exposure to ketamine at a concentration of 100 μM 1 hour prior to stimulation with vancomycin-exposed LAC or MW2. We found that the magnitude of suppression of TNF secretion was similar at all times studied (Figure 7).

## Discussion

We found that exposure of murine macrophages to ketamine inhibited TNF secretion by 18-34% after stimulation with CA-MRSA bacteria in the presence of antibiotics. The magnitude of the effect was comparable in response to both MW2 (USA400) and LAC (USA300) bacteria and was similar in the presence of either vancomycin (a lytic antibiotic associated with a greater TNF response to the bacteria) or daptomycin (a non-lytic antibiotic associated with a blunted TNF response to the bacteria). Our data suggest that ketamine administration to macrophages stimulated by CA-MRSA is associated with blunting of the TNF response to these virulent pathogens, and suggest that these findings may have therapeutic significance in MRSA sepsis. Furthermore, these data confirm and extend our previous observations that CA-MRSA bacteria exposed to daptomycin (versus vancomycin) trigger less TNF secretion by macrophages. The potentially beneficial anti-inflammatory effects of daptomycin and ketamine were additive (Figures 2, 3).

An improved understanding of the pathogenesis of sepsis and other life-threatening infections caused by CA-MRSA bacteria could expedite the development of novel strategies for the diagnosis, treatment, and/or prevention of these serious infections. CA-MRSA infections often are associated with severe and prolonged host inflammatory responses [43-46]. Prompt antibiotic treatment of these and other serious bacterial infections is indicated, but paradoxically has the potential to trigger excessive release of bacterial products and the subsequent augmentation of the host inflammatory response [15,16]. Macrophages are important sources of many of the pro-inflammatory cytokines (including IL-1β, IL-6, IL-8, IL-12, and TNF) secreted in response to staphylococci and other Gram-positive bacteria [15,16,41]. Although the cytokine cascade is essential for normal host defense, excessive or inappropriate inflammation can be harmful. Therefore we need an improved understanding of these interactions in order to develop better adjunctive therapies for patients with severe bacterial infections.

In a previous study, we found that exposure of either of two CA-MRSA strains isolated from Memphis children (or any of four other *S. aureus *isolates from children with invasive staphylococcal infections) to daptomycin (compared with vancomycin) led to a less pronounced macrophage inflammatory response, characterized by diminished secretion of TNF and reduced accumulation of the inducible nitric oxide synthase (iNOS) [11]. In this study, we found that this differential effect of daptomycin (versus vancomycin) was also observed when macrophages were stimulated with either of the two prototypical CA-MRSA strains most widely studied today: the USA400 isolate, MW2, and the USA300 isolate, LAC. Importantly, ketamine pre-incubation inhibited macrophage TNF secretion in response to both CA-MRSA strains in the presence of daptomycin as well as in the presence of vancomycin, and the greatest suppression of TNF secretion was noted in the presence of both daptomycin and ketamine.

The mechanism(s) responsible for the anti-inflammatory properties of ketamine are not known, but its neurological and psychotropic actions are believed primarily to be mediated by antagonism of NMDA receptors [21,47]. Glutamate is the brain's primary excitatory neurotransmitter. NMDA receptors are found in many cell types, including blood lymphocytes, lung macrophages, and multiple hematopoietic precursors in bone marrow cells [40,42,47,48]. Both ketamine and the chemically unrelated anticonvulsant dizocilpine (MK-801) are non-competitive antagonists of the NMDA receptor, one of the three known glutamate receptors [32,33,47]. APV is a competitive inhibitor of the classical NMDA receptor and acts on the NR2 component of the receptor (30, 33).

We found that MK-801 and APV also inhibited macrophage TNF secretion in response to antibiotic-treated MW2 or LAC cells. The magnitude of the inhibition by MK-801 (approximately 30%) and APV (25-35%) was comparable to that observed with ketamine (18-34%), and combinations of MK-801 and ketamine or of APV and ketamine did not exhibit additive or synergistic inhibition of TNF secretion. Furthermore, adding NMDA led to augmented macrophage TNF secretion in response to antibiotic-treated CA-MRSA bacteria, and the NMDA receptor antagonist, APV, blocked this effect. The suppression of TNF induced by ketamine was observed across a range of concentrations and throughout the incubation period.

Our study has its limitations. To translate the present findings, we are currently working on a clinical model to assess the clinical significance of ketamine's anti-inflammatory effects in patients with bacterial sepsis. Although studies of the effect of ketamine on macrophage responses to purified bacterial components such as Gram-negative lipopolysaccharide (LPS) or Gram-positive lipoteichoic acid (LTA) are instructive [23,24,49], we argue that characterization of the macrophage responses to whole organisms is more likely to provide clinical insights. Indeed, the pioneering experiments of Carswell and Old that identified TNF used whole bacteria as stimuli in macrophage sepsis simulation settings [49], and we have previously demonstrated that macrophage responses to live, antibiotic-treated staphylococci serve as a powerful model system. Furthermore, the model examines the effect of ketamine only in the presence of antibiotics (either vancomycin or daptomycin). In practice, this is a common clinical scenario. Our data suggest that clinically achievable concentrations of both ketamine and daptomycin could potentially inhibit the excessive macrophage inflammatory response that is observed in patients with severe staphylococcal infections.

## Conclusions

In the battle of sepsis everything counts. Adjunctive therapies of sepsis are greatly needed. Studies in animal models and clinical trials will be required to determine whether the anti-inflammatory effects of ketamine and/or other agents that block NMDA receptors could be beneficial in the treatment of severe staphylococcal infections.

## Abbreviations

CA-MRSA: community-associated methicillin-resistant *Staphylococcus aureus*; NMDA: N-methyl-D-aspartate; HA-MRSA: hospital-associated methicillin-resistant *Staphylococcus aureus*; iNOS: nitric oxide synthase; LPS: lipopolysaccharide; LTA: lipoteichoic acid; PVL: Panton-Valentine leukocidin; PSM: phenol-soluble modulins; APV: D-2-amino-5-phosphono-valerate; MK-801: dizocilpine; CFU: colony forming units; IL 1-12: interleukin 1-12; Le Bonheur Children's Hospital (LBCH)

## Competing interests

The authors declare that they have no competing interests.

## Authors' contributions

TS, RKHS, CAA, EM, LL and BSW contributed to acquisition of data. MSR was the medication consultant. TS, EM and BKE contributed to the conception and design and interpretation of the data. TS contributed to the statistical analysis. TS, EM and BKE gave the final approval.

## Presented in part at

National Society of Critical Care meeting in Nashville, TN, Feb. 2009

Southern Society of Clinical Investigation/American Federation for Medical Research meeting in New Orleans, LA, Feb. 2009

Pediatric Academic Societies' research meeting in Baltimore, MD, May 2009

American Academy of Pediatrics Section of Critical Care, Washington DC, October 2009
